# Characterization of aging tumor microenvironment with drawing implications in predicting the prognosis and immunotherapy response in low-grade gliomas

**DOI:** 10.1038/s41598-022-09549-3

**Published:** 2022-03-31

**Authors:** Zijian Zhou, JinHong Wei, Wenbo Jiang

**Affiliations:** 1 Department of Neurosurgery, Qingdao Municipal Hospital, Qingdao University, No.1 Jiaozhou Road, Qingdao, 266011 China; 2 School of Basic Medical Sciences, Southwest Medical University, Luzhou, 646000 China

**Keywords:** Cancer, Computational biology and bioinformatics, Genetics, Immunology, Molecular biology, Neuroscience, Biomarkers, Diseases, Medical research, Molecular medicine, Oncology, Risk factors

## Abstract

Aging tumor microenvironment (aging TME) is emerging as a hot spot in cancer research for its significant roles in regulation of tumor progression and tumor immune response. The immune and stromal scores of low-grade gliomas (LGGs) from TCGA and CGGA databases were determined by using ESTIMATE algorithm. Differentially expressed genes (DEGs) between high and low immune/stromal score groups were identified. Subsequently, weighted gene co-expression network analysis (WGCNA) was conducted to screen out aging TME related signature (ATMERS). Based on the expression patterns of ATMERS, LGGs were classified into two clusters with distinct prognosis via consensus clustering method. Afterwards, the aging TME score for each sample was calculated via gene set variation analysis (GSVA). Furthermore, TME components were quantified by MCP counter and CIBERSORT algorithm. The potential response to immunotherapy was evaluated by Tumor Immune Dysfunction and Exclusion analysis. We found that LGG patients with high aging TME scores showed poor prognosis, exhibited an immunosuppressive phenotype and were less likely to respond to immunotherapy compared to those with low scores. The predictive performance of aging TME score was verified in three external datasets. Finally, the expression of ATMERS in LGGs was confirmed at protein level through the Human Protein Atlas website and western blot analysis. This novel aging TME-based scoring system provided a robust biomarker for predicting the prognosis and immunotherapy response in LGGs.

## Introduction

Low-grade gliomas (LGGs) which are subdivided into oligodendroglioma, astrocytoma and oligoastrocytoma, represent a group of primary tumors originating from glial cells in the central nervous system and are very common in young adults^1^. Despite the widely-accepted notion that LGGs exhibit inertia in histological malignancy, they account for approximately 20% of all primary intracranial tumors and the prognosis of LGG patients can be highly variable, with the median overall survival ranging from 5.6 to 13.3 years^2–4^. The clinical outcomes for LGG patients are far away from satisfactory even though maximum surgical resection combined with postoperative chemotherapy and radiotherapy are applied^5^. Therefore, investigation of the underlying mechanism in tumorigenesis and tumor progression is urgently needed, with drawing implications in predicting the prognosis and exploring novel treatment for patients suffering from LGGs.

Cancer has been recognized as a type of age-related disease, which mostly attributed to the fact that many cancers arise as we age^6^. An accumulating number of studies have demonstrated that some common features are shared between aging progress and development of tumor, in which cellular senescence is considered to profoundly affect the physiological and pathological processes^7,8^. As a dynamic evolving environment, tumor microenvironment (TME) which refers to the surrounding compositions around tumor cells, includes a series of immune cells, stromal cells and cytokines and plays substantial roles in tumor progression and metastasis^9^. Previous studies reveal that TME appears susceptible to the impact of aging progress, especially the involved fibroblasts and immune cells. Moreover, the age-induced changes of these components in TME are considered to play a crucial role in tumor progression, which is significantly associated with prognosis^10^. While senescent fibroblasts are proved to promote the proliferation of epithelial tumor cells in immunocompromised mice^11^, tumor aggressiveness may not correlate with age in all types of tumors. The complex interrelationship between aging TME and development of tumor need to be further exemplified^10^. The astounding success obtained in the clinical trials of immunotherapy has shed light on the treatment of cancers. For example, immune checkpoint blockade (ICB) therapy (anti-CLTA-4, anti-PD1 and anti-PD-L1) targeting T cells can improve anti-tumor immunity and exert durable clinical benefit in patients. Unfortunately, the majority of patients get no or minimal clinical benefit due to the lack of precise selection^12^. In recent years, the understanding of immune contexts of TME has advanced, which contributes to the identification of multiple classifications of patients based on TME for predicting and guiding immunotherapeutic responsiveness^13^. However, the diverse roles of aging TME in immunotherapy have not been well documented.

In this study, we developed an aging TME related signature (ATMERS) in LGGs through comprehensive analysis of transcriptomic data from TCGA and CGGA databases. Furthermore, aging TME scoring system based on ATMERS was established to predict the prognosis and immunotherapeutic response for LGG patients. Furthermore, external datasets were used to verify the performance of aging TME score which serves as an independent predictor. Finally, western blot analysis was performed to validate the ATMERS at protein level.

## Results

### Identification of aging TME related signature

**Figure 1:**
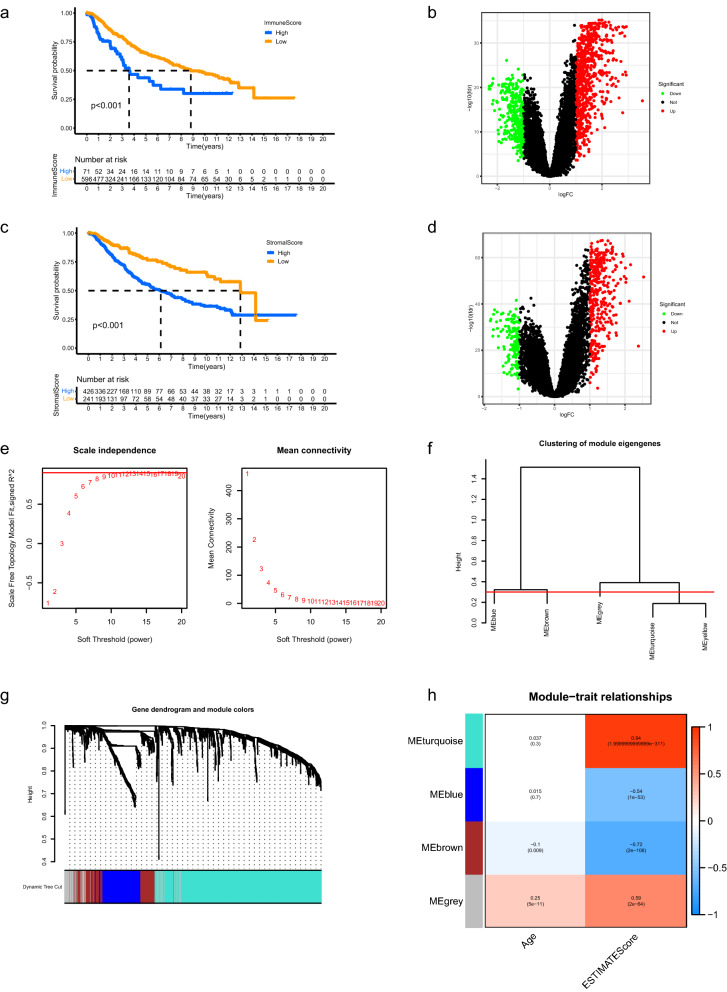
Identification of aging TME related signature. (**a**) Kaplan–Meier analysis revealed that the overall survival of LGG patients in high immune score group was shorter than those in low score group. (**b**) Volcano plots of DEGs between low and high immune score groups. The red dots represented upregulated genes and the green dots represented downregulated genes. The black dots represented genes with no significant difference. (**c**,**d**) Similar results were obtained between low and high stromal score groups. (**e**) Determination of the scale-independence degree (left) and the mean connectivity index (right) for soft-threshold values ranging from 1 to 20. (**f**) Clustering for the module eigengenes. The cut height was set at 0.30 depicted with the red line. (**g**) Dendrogram of all DEGs and modules with different colors. (**h**) Heatmap showing the key modules which mostly correlated with age and ESTIMATE scores of LGGs. The Pearson correlation coefficients and p values were displayed in cells. TME, tumor microenvironment; LGG, low-grade glioma; DEGs, differentially expressed genes.

The corresponding clinicopathological information for LGG samples of the two datasets was shown in Supplementary Table 1 and Supplementary Table 2, respectively. 667 LGG patients in the merged dataset were classified into low and high immune score groups according to the immune scores and survival information. As shown in Fig. 1a, patients with high immune scores lived significantly shorter than patients with low scores (p < 0.001). The robust DEGs between low and high immune score groups were displayed in Fig. 1b. Similar results were obtained between low and high stromal score groups (Fig. 1c,d, p < 0.001). The robust DEGs above were merged for further analysis. Weighted Gene Co-expression Network Analysis (WGCNA) was performed to determine the key module eigengenes which significantly correlated with aging TME based on the expression profiles of extracted DEGs. The soft threshold (power) value was set at 10 (scale independence R^2^ = 0.86, mean connectivity = 8.49) and the cut height was set at 0.30. We found a total of four co-expression modules (Fig. 1e–h), in which the brown module exhibited negative correlation with aging TME (R^2^ = − 0.1 and p = 0.009 with age, R^2^ = − 0.72 and p = 2e−108 with ESTIMATE score) and the grey module demonstrated positive correlation with aging TME (R^2^ = 0.25 and p = 5e−11 with age, R^2^ = − 0.59 and p = 2e−64 with ESTIMATE score). The two module eigengenes were merged and a total of 241 genes were obtained and regarded as ATMERS (Supplementary Table 3).

### Classification for LGG samples

**Figure 2:**
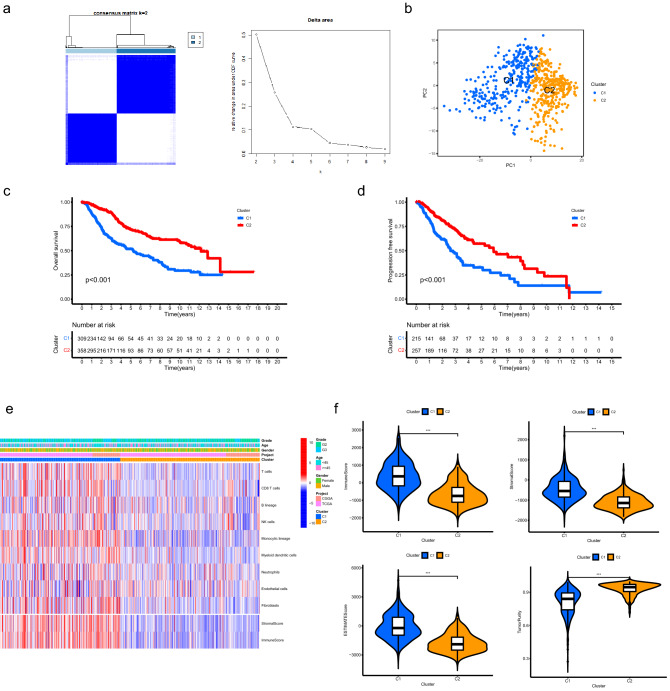
Classification for LGG samples based on the expression patterns of ATMERS. (**a**) Consensus clustering for LGG samples identified two clusters. (**b**) PCA verified the subgroup assignment of LGG samples. (**c**) C1 exhibited significantly shorter overall survival than C2. (**d**) The progression free survival for C1 was significantly shorter than C2. (**e**) Heatmap showing the distinct TME patterns between C1 and C2. (**f**) Comparisons of immune score, stromal score, ESTIMATE score and tumor purity between C1 and C2. * means p < 0.05, ** means p < 0.01, and ***means p < 0.001. LGG, low-grade glioma; ATMERS, aging tumor microenvironment related signature; PCA, principal component analysis; TME, tumor microenvironment.

Based on the expression patterns of ATMERS, LGG patients were classified into two clusters (Fig. 2a). Principal component analysis (PCA) verified the subgroup assignment of LGG samples (Fig. 2b). Kaplan–Meier survival analysis revealed that C1 exhibited significantly shorter overall survival than C2 (Fig. 2c, p < 0.001) and the progression free survival for C1 was significantly shorter than C2 (Fig. 2d, p < 0.001), indicating the prognosis for patients in C1 was worse than those in C2. The MCP counter analysis demonstrated that most of the crucial immune and stromal cells in the TME of C1 were upregulated than those in C2, especially T cells, CD8 T cells, Monocytic lineage, Myeloid dendritic cells and Fibroblasts (Fig. 2e, Supplementary Fig. 1). As depicted in Fig. 2f, the C1 presented significantly higher immune, stromal, and ESTIMATE score than C2 (p < 0.001). We found lower tumor purity in C1 compared to C2 (p < 0.001).

### Comparison of the prognosis between low and high Aging TME score groups

Univariate cox regression analysis was carried out to determine favorable and unfavorable ATMERS which positively or negatively correlated with the prognosis of LGG patients. Consistent with the above results, the GSVA (gene set variation analysis) scores for favorable ATMERS of C1 were significantly lower compared to C2 and the GSVA scores for unfavorable ATMERS of C1 were significantly higher (Supplementary Fig. 2A). The expression levels for favorable ATMERS of C1 were lower than those of C2 and the expression levels for unfavorable ATMERS of C1 were higher compared to C2 (Supplementary Fig. 2B).

**Figure 3:**
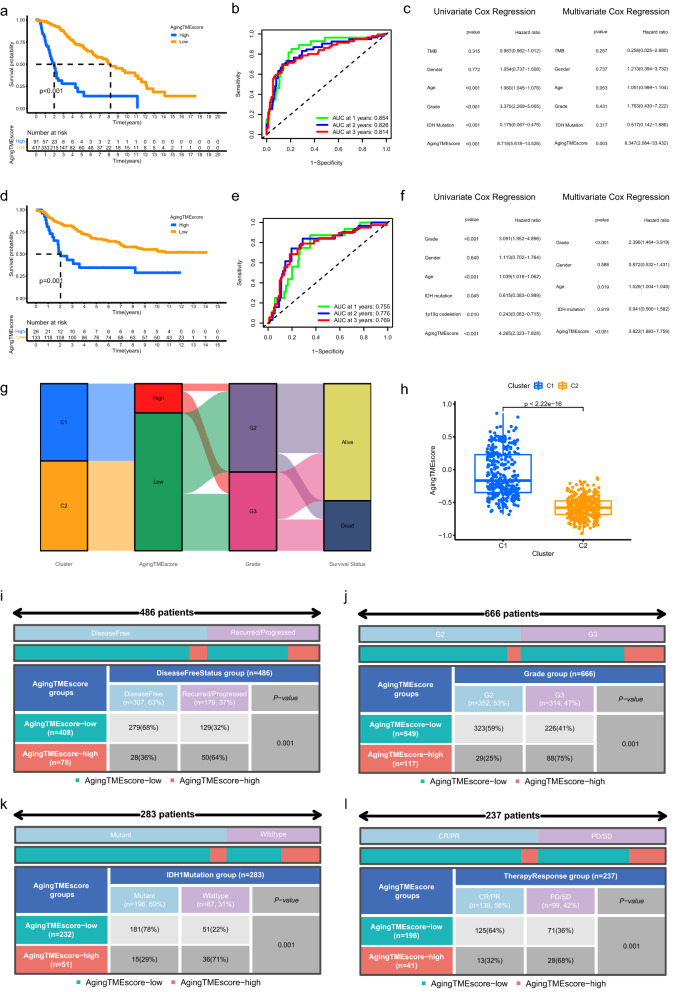
Comparison of the prognosis between low and high Aging TME score groups. (**a**–**f**) Kaplan–Meier survival analysis, time-dependent ROC curves and univariate/multivariate cox regression analysis of aging TME score in TCGA cohort (**a**–**c**) and CGGA cohort (**d**–**f**), respectively. (**g**) Alluvial diagram of clusters, aging TME score groups, grade and survival status. (**h**) Comparisons of aging TME scores between C1 and C2. (**i**–**l**) Correlation between aging TME score and disease free status (**i**), grade (**j**), IDH1 mutation status (**k**), conventional therapy response (**l**). LGG, low-grade glioma; ROC, receiver operating characteristic; AUC, area under curves; TME, tumor microenvironment.

LGG patients were separated into low and high aging TME score groups according to the scoring system based on GSVA method. Kaplan–Meier survival analysis indicated that high aging TME score group exhibited significantly shorter overall survival than low aging TME score group in LGG patients from TCGA database (Fig. 3a, p < 0.001). The AUC (area under curves) values for the ROC (Receiver Operating Characteristic) curves at 1, 2, 3 years were 0.854, 0.826 and 0.814, respectively, demonstrating that the predictive accuracy was pretty well (Fig. 3b). Univariate cox regression analysis revealed that the aging TME score significantly correlated with prognosis (Fig. 3c). Multivariate cox regression analysis revealed that the aging TME score served as an independent factor for predicting the prognosis of LGG patients in the TCGA cohort (Fig. 3c, both values were < 0.005). Similar results were obtained in LGG patients from CGGA database (Fig. 3d–f). In addition, we found that the aging TME scores for younger patients were significantly lower compared to older patients and the aging TME scores of patients with grade G2 were lower than those of patients with grade G3 (Supplementary Fig. 3A). Stratified analysis was performed to further confirm the prognostic value of the aging score. For example, LGG patients were divided into young age group (age < 45) and old age group (age ≥ 45). The young age group was further classified into low and high aging TME score groups. We found that LGG patients with low aging TME scores presented better prognosis either in the young or old age group (Supplementary Fig. 3B). Similar results were acquired when LGG patients were further stratified according to gender or grade (Supplementary Fig. 3B).

The alluvial diagram (Fig. 3g) exhibited the distribution of LGG patients across clusters, aging TME score groups, grades and survival status. Moreover, the aging TME scores for C1 were significantly higher than C2 (Fig. 3h). The proportion of LGG patients with disease free status in the low aging TME score group was substantially higher than those in the high aging TME score group (Fig. 3i, p < 0.001). The grade for LGGs in the low aging TME score group was lower (Fig. 3j, p < 0.001). We found more *IDH1* mutant LGG samples in the low aging TME score group (Fig. 3k, p < 0.001). LGG patients in the low aging TME score group were more sensitive to conventional treatment (Fig. 3l, p < 0.001). All these findings indicated that LGG patients with low aging TME scores were more likely to get better prognosis.

### Construction of nomogram model

**Figure 4:**
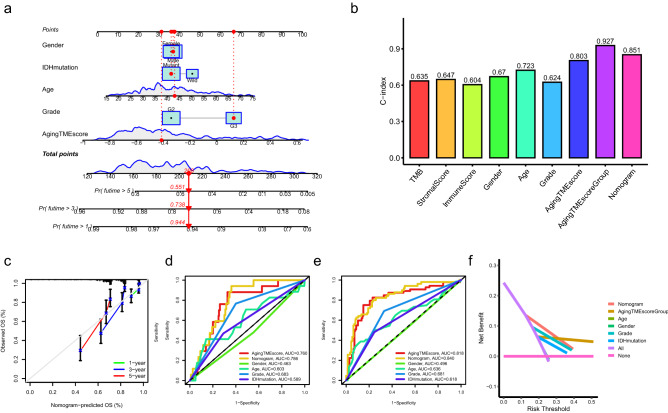
Construction of nomogram model. (**a**) Nomogram model was constructed combining the aging TME score and multiple clinicopathological factors. (**b**) Determination of C-index values of multiple clinicopathological factors. (**c**) Calibration curves for the nomogram model. (**d**,**e**) ROC curves for the nomogram model and other clinicopathological factors at the time of 1 (**d**) and 2 (**e**) years. (**f**) DCA for the nomogram model and other clinicopathological factors. TME, tumor microenvironment; C-index, consistency index; ROC, receiver operating characteristic; AUC, area under curves; DCA, decision curve analysis.

For clinical practice, a nomogram prognostic model combining aging TME score and other clinicopathological factors was constructed to improve the predictive performance (Fig. 4a). The nomogram was tested by proportional hazard assumption, in which p values for all the variables in the nomogram model were more than 0.05 (Supplementary Table 4). As depicted in Fig. 4b, the C-index (consistency index) value with 0.927 for aging TME score group (high or low) showed the highest compared to other clinicopathological factors. The C-index value for nomogram model was 0.851, which were higher than other factors. The calibration curves for the nomogram model indicated good agreement between the predictive values and the actual observations (Fig. 4c). Regarding the ROC curves, the AUC values of the nomogram for predicting 1- and 3-year overall survival were 0.786 and 0.840, respectively, which were higher than those of other factors (Fig. 4d,e). The results of DCA (decision curve analysis) for the nomogram model confirmed its outstanding performance for predicting the prognosis (Fig. 4f, 3-year overall survival). Despite the fact that LGG patients with distinct immune or stromal scores tended to have different prognosis (Fig. 1a,c), as shown in the ROC curves (Supplementary Fig. 4), the AUC values of the nomogram model or aging TME score were evidently higher than those of immune or stromal score when predicting the 1, 2, and 3-year overall survival. Moreover, the results of DCA of the nomogram model and aging TME score further confirmed their robust performance for predicting the prognosis, compared to immune or stromal score (Supplementary Fig. 4).

### Functional enrichment analysis

**Figure 5:**
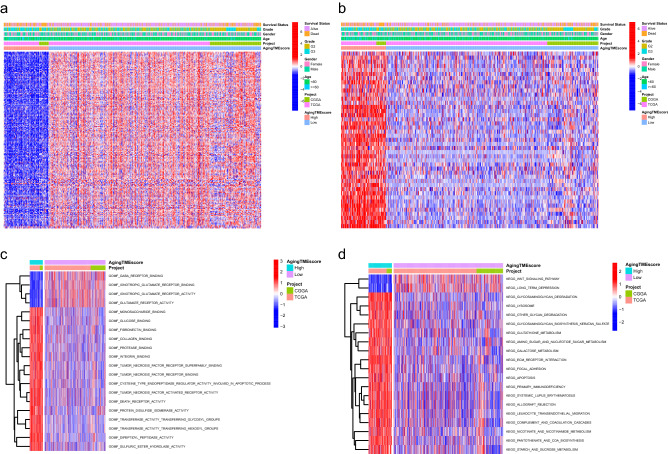
Functional enrichment analysis between the low and high aging TME score groups. (**a**) The expression patterns of favorable ATMERS between groups. (**b**) The expression patterns of unfavorable ATMERS between groups. (**c**) The enrichment analysis of GO molecular functions between groups. (**d**) The enrichment analysis of KEGG pathways between groups. TME, tumor microenvironment; ATMERS, aging tumor microenvironment related signature; GO, Gene Ontology; KEGG, Kyoto Encyclopedia of Genes and Genomes.

The expression levels for favorable ATMERS of the high aging TME score group were lower than those of the low aging TME score group and the expression levels for unfavorable ATMERS between the two groups exhibited reverse patterns (Fig. 5a,b). Molecular functions concerning programed cell death, including tumor necrosis factor receptor superfamily binding, tumor necrosis factor receptor binding, tumor necrosis factor activated receptor activity and death receptor activity, were significantly enriched in the high aging TME score group (Fig. 5c). Moreover, KEGG (Kyoto Encyclopedia of Genes and Genomes) pathways related to tumorigenesis or progression, such as ECM (extracellular matrix) receptor interaction, focal adhesion and apoptosis, were substantially enriched in the high aging TME score group compared to the low score group (Fig. 5d).

### Immunosuppressive phenotype was identified in the TME of the high aging TME score group

**Figure 6:**
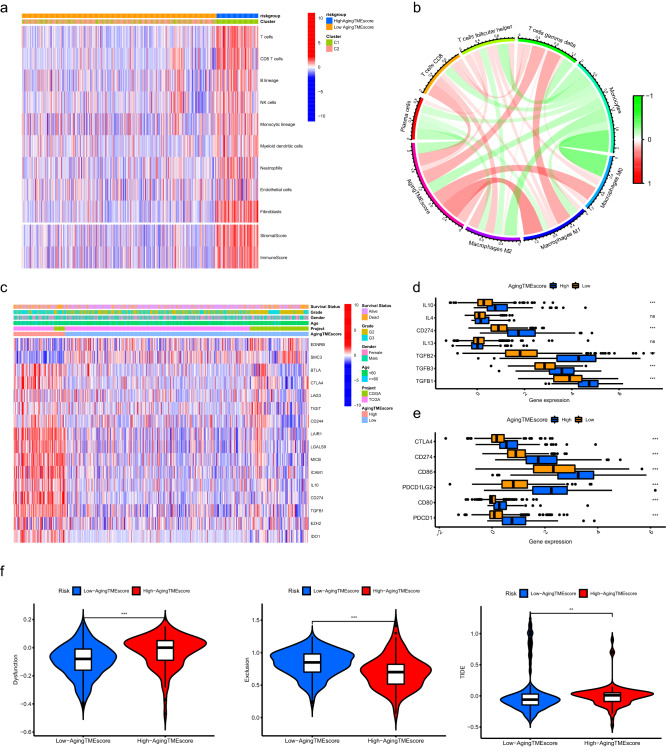
Immunosuppressive phenotype was identified in the TME of the high aging TME score group. (**a**) Results of the abundance of immune or stromal cells calculated by MCP counter algorithm and immune or stromal scores calculated by ESTIMATE algorithm. (**b**) The correlation between aging TME score and infiltrated immune cells evaluated by CIBERSORT algorithm. (**c**) The expression profiles of genes involved in the negative regulation of anti-tumor immune response between low and high aging TME score groups. (**d**) Differentially expression of the immune suppressive cytokines between the two groups. (**e**) Differentially expression of the common immune checkpoints between the two groups. (**f**) Comparisons of TIDE scores between the two groups. * means p < 0.05, ** means p < 0.01, and ***means p < 0.001. TME, tumor microenvironment.

As shown in Fig. 6a, the abundance of most of the immune or stromal cells for the high aging TME score group was substantially higher compared to the low score group, which was consistent with the results of immune or stromal scores calculated by ESTIMATE algorithm (Supplementary Fig. 5A). CIBERSORT algorithm was utilized to further explore the correlation between aging TME score and infiltrated immune cells in TME. We found that the aging TME score positively correlated with the infiltrated immune suppressive cells such as T follicular helper cells and macrophages, indicating an immunosuppressive phenotype in the TME (Fig. 6b, Supplementary Fig. 5B). Furthermore, we explored the immune molecules negatively regulating the anti-tumor immune response to confirm the immunosuppressive phenotype in the high aging TME score group. The immune related genes negatively regulating The Cancer-Immunity Cycle were obtained from Tracking Tumor Immunophenotype website (http://biocc.hrbmu.edu.cn/) and the expression profiles of these genes were identified between the low and high score groups. Most of these genes were highly expressed in the high aging TME score group while the expression levels of *EDNRB* and *SMC3* were higher in the low score group (Fig. 6c, Supplementary Fig. 5C). The expression levels of chemokines induced by immune suppressive cells, such as *IL10*, *CD274* (*PD-L1*), *TGFB1*, *TGFB2* and *TGFB3* were also significantly elevated in the high score group^14–16^ (Fig. 6d). In addition, common immune checkpoints including *PDCD1, CD274, PDCD1LG2, CTLA4, CD80* and *CD86* were upregulated in the high score group (Fig. 6e). These findings indicated that LGG patients in the high aging TME score group exhibited suppressive antitumor immunities, which might contribute to their pessimistic prognosis. TIDE (Tumor Immune Dysfunction and Exclusion) website was used to explore the immunotherapy response. We found that the immune dysfunction scores for the low aging TME score group were significantly lower while the immune exclusion scores were higher compared to the high score group. Importantly, the TIDE scores were substantially lower in the low aging TME score group, suggesting the LGG patients with low aging TME scores were more sensitive to immunotherapy (Fig. 6f).

### Somatic mutation analysis between the low and high aging TME score groups

**Figure 7:**
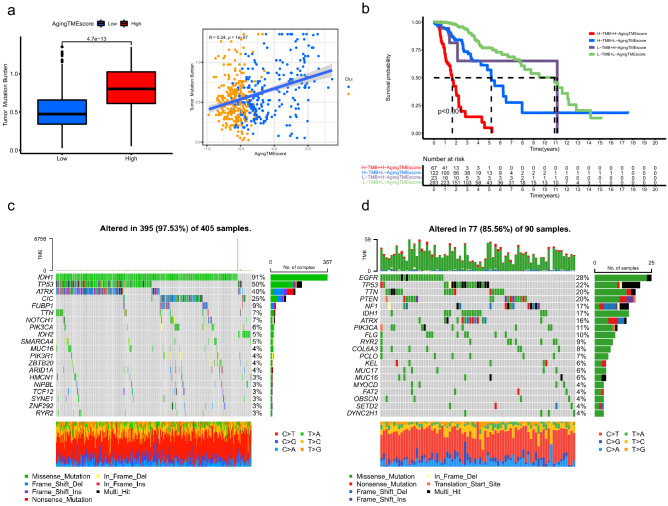
Somatic mutation analysis between the low and high aging TME score groups. (**a**) Correlation analysis between aging TME score and TMB. (**b**) Kaplan–Meier survival analysis of aging TME score and TMB. (**c**,**d**) Top 20 mutated genes in the low (**c**) and high (**d**) aging TME score groups. TME, tumor microenvironment; TMB, tumor mutation burden.

The TMBs (tumor mutation burdens) of the high TME score group were significantly higher than those of the low score group (p = 4.1e−13) and we found that the aging TME score positively correlated with the TMBs (R = 0.24, p = 1e−07) (Fig. 7a). Kaplan–Meier survival analysis demonstrated that LGG patients with high TMBs and high aging TME scores presented the worst prognosis and patients with low TMBs and low aging TME scores got the highest survival rate. Moreover, LGG patients with low TMBs and high aging TME scores tended to get worse prognosis than those with high TMBs and low aging TME scores (p < 0.001, Fig. 7b). All these data suggested that TMB negatively correlated with the prognosis and aging TME score served as an independent predictor for prognosis regardless of TMB. As depicted in Fig. 7c,d, the top 5 genes with the highest mutation frequency in low aging TME score group were *IDH1, TP53, ATRX, CIC* and *FUBP1*, whereas, *EGFR, TP53, TTN, PTEN* and *NF1* were included in high aging TME score group. Hao demonstrated that, based on the TCGA data, the top six most frequently mutated genes in LGGs were *IDH1* (77.25%), *TP53* (48.04%), *ATRX* (39.22%), *CIC* (22.75%), *TTN* (17.06%), and *PIK3CA* (8.43%), while the top six most frequently mutated genes in GBMs (glioblastoma multiforme) were *PTEN* (34.86%), *TTN* (32.57%), *TP53* (31.55%), *EGFR* (26.97%), *MUC16* (18.07%) and *NF1* (12.98%)^17^. This indicated that the top 5 most mutated genes in high Aging TME score group including *EGFR, TP53, TTN, PTEN and NF1*, were also frequently mutated in GBMs, implying that the LGGs of high Aging TME score group might represent more aggressive tumors genetically similar to GBMs. Consistent with our results, gene mutations including *EGFR, TP53* and *PTEN* have been reported to significantly correlate with poor survival in gliomas^18–22^.

### Validation of ATMERS

**Figure 8:**
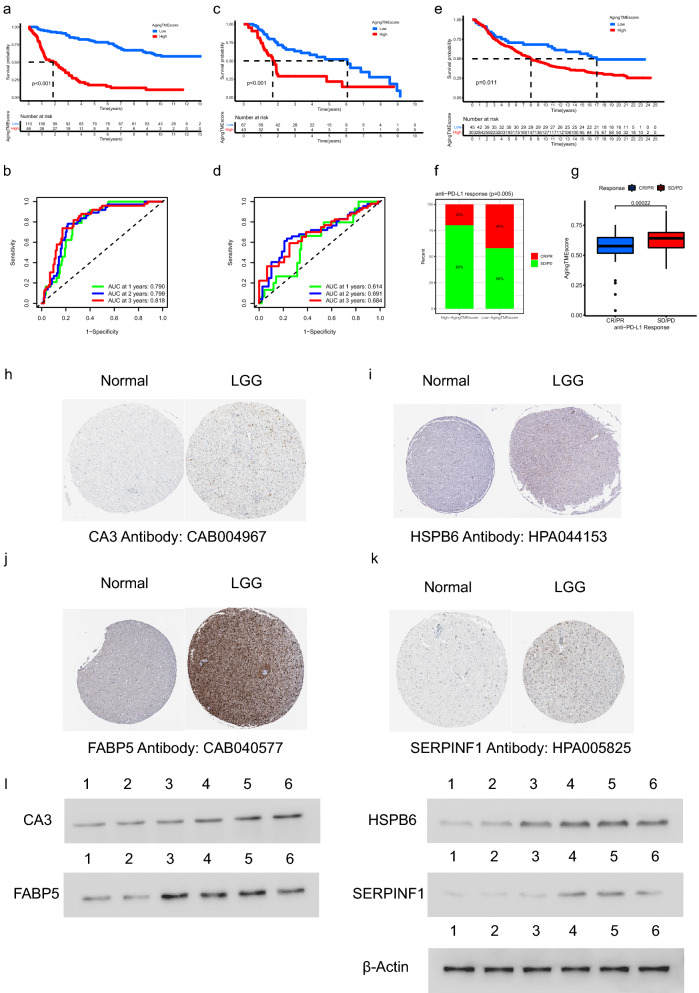
Validation of ATMERS. (**a**,**b**) Kaplan–Meier survival analysis (**a**) and time-dependent ROC curves (**b**) of aging TME score in the validation cohort from CGGA database (DataSet ID: mRNAseq_325). (**c**,**d**) Similar results obtained in the merged validation cohort from GEO database (GSE4271, GSE4412, GSE43378 and GSE84010). (**e**) Kaplan–Meier survival analysis between low and high aging TME score groups in IMvigor210 cohort. (**f**) Proportion of anti-PD-L1 therapeutic response in low and high aging TME score groups in IMvigor210 cohort. (**g**) Comparison of ageing TME scores between patients with different anti-PD-L1 therapeutic response in IMvigor210 cohort. (**h**–**k**) Identification of unfavorable ATMERS in immunohistochemistry staining. (**l**) Determination of unfavorable ATMERS using western blot analysis in which lane 1 represented normal brain tissues, lane 2, 3 represented LGG tissues in grade II, lane 4, 5 and 6 represented LGG tissues in grade III. ATMERS, aging tumor microenvironment related signature; ROC, receiver operating characteristic; AUC, area under curves; TME, tumor microenvironment; LGG, low-grade glioma, CR/PR: complete response/partial response; PD/SD: progressed/stable disease.

In the validation cohort from CGGA database (DataSet ID: mRNAseq_325), LGG patients in the high aging TME score group got worse prognosis compared to the low score group (p < 0.001, Fig. 8a). The AUC values for the time-dependent ROC curves at 1, 2, 3 years were 0.790, 0.799 and 0.818, which confirmed the predictive accuracy of ATMERS (Fig. 8b). Similar results were obtained in the merged validation cohort from GEO database (GSE4271, GSE4412, GSE43378 and GSE84010), with the predictive accuracy of 0.614, 0.691 and 0.684 at 1, 2, 3 years. (Fig. 8c,d). Furthermore, the predictive value of aging TME score derived from ATMERS was verified in IMvigor210 cohort (p < 0.001, Fig. 8e). We found that patients with low aging TME scores tended to get better response to anti-PD-L1 therapy (p = 0.005, Fig. 8f) and the aging TME scores of patients sensitive to anti-PD-L1 immune therapy were significantly lower compared to patients resistant to the immune therapy (p = 0.00022, Fig. 8g). Considering that urothelial carcinoma in IMvigor210 cohort represented a distinct form of cancer, we demonstrated that the TME of LGG shared common features with those of urothelial carcinoma regarding the expression patterns of TME related genes (Supplementary Fig. 6). By using WGCNA, we found that 229 out of 241 genes in the ATMERS which were identified in LGGs, significantly correlated with the TME of urothelial carcinomas. Consistent with the results in LGGs, the high aging TME score group exhibited significantly higher scores with respect to TME, compared to the low aging TME score group in IMvigor210 cohort. Based on these findings, we suppose that there are major similarities in TME and aging TME between LGG and urothelial carcinoma. Therefore, the ATMERS based aging TME scoring system established in LGG can be reasonably applied to urothelial carcinoma.

As depicted in Fig. 8h–k, four genes including *CA3, HSPB6, FABP5 and SERPINF1* were randomly selected from the unfavorable ATMERS and scanned on The Human Protein Atlas website. The expression levels of the corresponding proteins were found upregulated in LGG tissues compared to normal brain tissues. Moreover, western blot analysis was carried out to further confirm the high expression patterns of the four genes in LGG tissues at protein level (Fig. 8l, the original blots are presented in Supplementary Fig. 7).

## Discussion

Despite the WHO (World Health Organization) has published a renewed classification method for gliomas by integrating histopathological results with molecular phenotypes such as *IDH* mutation and 1p/19q codeletion^23^, patients with LGGs show variable clinical prognosis even with the same diagnosis due to high heterogeneity of tumors^24^. Besides, current therapy strategy including surgery, radiotherapy and chemotherapy could not significantly improve the poor prognosis of LGG patients. It remains challenging for the treatment of LGGs^25^. As a crucial component of tumor, TME is recognized as a dynamic and heterogeneous environment and closely correlates with tumor initiation and progression^13^. Novel immunotherapy targeting TME such as ICB therapy, has achieved astounding successes across diverse tumor types^26–29^. Numerous studies have revealed the indispensable roles of TME in regulation of tumor immune responses and immunotherapy response^13^. The realization of the essential roles of TME has revolutionized our understanding of tumor. Many studies have been focusing on the local and systemic microenvironment rather than tumor cell only. In recent years, it is well documented that the essential populations within TME are susceptible to age-related impact. The significant roles of aging TME in the regulation of tumor progression and tumor immune response need to be extensively addressed. Paradoxically, the senescent cells and the induced senescence-associated secretory phenotype within TME regulate tumor development in both tumor-suppressive and tumor-promoting ways^30,31^. Therefore, in-depth investigation of aging TME will aid in our understanding of the complexity and diversity of the development of tumor with appealing implications in predicting the prognosis and immunotherapy response for patients with tumor.

Despite the fact that Cheng et al. have revealed ten aging-related genes which serve as potential prognostic biomarkers for patients with gliomas^32^, there are limited studies focusing on the exploration of aging TME, especially for LGGs. Unlike the aging-related prognostic model which was demonstrated by Cheng et al.^32^, including *EEF2, ARNTL, FBXO4, CHEK1, CHEK2, CTSC, MBD2, HMGA2, IGFBP2* and *TIMP1*, we identified a total of 241 genes which were defined as ATMERS in LGGs through comprehensive analysis of RNA-seq data from TCGA and CGGA databases. To guide the genetic models in gliomas, Liu et al. screened out 29 highly overlapping genes with strong prognostic potential by comprehensively reviewing 138 prognostic models^33^, in which three genes were involved in the ATMERS developed in our study, including LGALS3, BMP2 and KCNB1. Furthermore, aging TME scoring system based on ATMERS was developed by using GSVA method to predict the prognosis and immunotherapeutic response of LGG patients. Finally, three independent external datasets from distinct databases were employed to verify the robust performance of aging TME score which served as an independent predictor. In addition, western blot analysis was carried out for the validation of ATMERS at protein level. We culminated in several consensuses based on the findings obtained in this study: (1) consistent with previous studies, we found that TME significantly correlated with the prognosis of LGG patients; (2) aging TME score served as a robust biomarker for the prognosis and therapeutic response to both conventional treatment and ICB immune therapy in LGGs; (3) LGGs with high aging TME scores tended to be determined as immunosuppressive phenotype; (4) LGGs with high aging TME scores tended to bear high TMBs.

Cellular senescence was firstly raised by Leonard Hayflflick in the 1960s. Senescent cells are arrested in a state of irreversible cell cycle after repeated rounds of replication and are resistant to cell death such as apoptosis, thus, they can continue to survive and accumulate as we age^34,35^. There are so many molecules involved in the formation and stabilization of senescence due to the complicated mechanisms. It is in a dilemma to accurately determine senescence-related genes. Therefore, we screened out a series of genes which were susceptible to age-related impact via WGCNA method and then identified ATMERS in combination with TME score. Specifically, in order to comprehensively characterize aging TME, our ATMERS included two groups of genes which were negatively or positively associated with age and TME, respectively.

We identified two molecular patterns (C1 and C2) based on the expression of ATMERS via consensus clustering method in LGGs. Interestingly, we found that all the LGG samples of C2 belonged to low aging TME group and the aging TME scores for C2 were significantly lower compared to C1, suggesting the consistency between consensus clustering method and aging TME scoring system. In addition, the distinct clinical outcomes between C1 and C2 indicated the underlying interrelationship between aging TME and the prognosis of LGG patients.

It is well known that aging TME is characterized by senescent cell and senescence-associated secretory phenotype (SASP)^31^. Fibroblasts account for the most common stromal cells within TME, and various soluble factors secreted by senescent fibroblasts play a crucial role in the regulation of migration and proliferation of tumor cells. For example, the extracellular matrix (ECM) cytokines secreted by aged dermal fibroblasts contribute to the decrease in collagen density, which can promote the aggression of melanoma cells^36^. Consistent with previous studies, functional analysis in our study demonstrated that ECM receptor interaction related KEGG pathway was significantly enriched in high aging TME score group. Moreover, Kaur et al. suggested that activation of the WNT signaling pathway induced melanocytes bypassing senescence^37^. Similarly, we found that the WNT signaling pathway was significantly enriched in low aging TME score group. Furthermore, we found significantly positive correlation between age and aging TME score (R = 0.13, p = 0.00085, Supplementary Fig. 8A). The age for LGG patients with high aging TME scores were substantially higher than those with low scores (p = 1.9e−11, Supplementary Fig. 8B). Moreover, an increasing number of evidence indicated that senescent immune cells played an important role in promoting the accumulation of senescent cells during aging^38^. It was reported that accumulated senescent cells contributed to the progression of many types of cancers^39^. In our study, we found that both the immune and stromal scores for high aging TME score group were significantly higher, indicating an increase in the immune or stromal components within the TME of high score group, which might attribute to the accumulation of senescent immune and stromal cells. Additionally, we acquired a total of 6 molecules including *IL6, AREG, CXCL12, TGFβ, VEGF* and *CCL2*, which can be upregulated upon senescence, influence immune cell functions, and play tumor-promoting roles in TME^30^. The expression levels of the corresponding genes were compared between low and high aging TME score groups. Consistent with the above results, the genes were highly expressed in high aging TME score group except for *CXCL12* (Supplementary Fig. 9). Collectively, all these findings suggested that the aging TME score built in our study not only had outstanding performance in predicting prognosis and immunotherapy response, but also could serve as an indicator to characterize and quantify the senescent status of TME for individuals to some extent. Considering the complexity and diversity of aging TME, we believe that extensive in vivo and in vitro experiments are needed to further prove it.

Studies have shown that age-induced immunosenescence usually occurs in immune cells involved in tumor immunity response, which could induce the infiltration of immunosuppressive cell types such as M2 tumor associated macrophages (TAMs), and result in the increased predisposition to tumor progression^40^. A large amount of evidence has pointed that M2-like immunosuppressive macrophages play a key role in promoting tumor progression in the aging context^41^. Consistent with the previous findings, our results demonstrated that LGGs in high aging TME score group presented an immunosuppressive phenotype with more infiltrating M2 macrophages and higher expression of immunosuppressive genes. In addition, Ladomersky et al. reported a robust increase in the expression of PD-L1 in older samples, indicating older patients with lymphoma, glioma and leukemia, may be less responsive to immunotherapy like ICB treatment^42,43^. Despite the fact that many conflicting studies were reported according to the published data^44^, we found that LGG patients with high aging TME scores were resistant to ICB immune therapy in our study.

There were still some limitations in our study. Firstly, the urothelial carcinomas in the validation dataset (IMvigor210 cohort) represented a distinct form of cancer compared to LGG, further investigation regarding in vivo and in vitro research would be needed to explore the correlation between the ATMERS identified in LGGs and the characteristics of aging TME in other forms of cancer. Secondly, due to limited information of the acquired data, only IDH1 mutation status, gender, age and grade were involved in the construction of the multivariate cox regression model and the nomogram model in our study. In the future, with the enrichment of the datasets with respect to multiple clinicopathological factors including IDH1 mutation status, 1p-19q codeletion status, tumor size, extent of tumor resection, a nomogram model based on multi-omics data would be built to predict the prognosis more efficiently. Thirdly, it might not be statistically reliable to conclude that the four randomly selected genes from unfavorable ATMERS were related to worse prognosis in LGGs, only based on the western blotting analysis of five samples, despite the fact that we have verified the prognostic values of the genes in RNA-seq data from different independent cohorts. In the future, we would collect more LGG samples to explore the correlation between gene expression at protein level and the prognosis of gliomas.

## Conclusion

We identified a gene signature which significantly correlated with aging TME, based on which we developed a robust aging TME scoring system to predict the prognosis and immunotherapeutic response of LGG patients. This novel score could also reflect the status of aging TME and reveal the close relationship between aging TME and immunosuppressive phenotype in LGGs. Our study might contribute to the understanding of aging TME and guide the development of aging TME-targeted therapy for LGGs in future.

## Methods

### Data acquisition

The RNA sequencing (RNA-seq) data for a total of 508 LGG samples were obtained from TCGA database (The Cancer Genome Atlas, http://cancergenome.nih.gov/). The corresponding annotation file, Genome Reference Consortium Human Build 38 (GRCh38), was downloaded from the Ensembl website (http://asia.ensembl.org/). The microarray dataset (dataset ID: mRNA-array_301) containing 159 LGG samples was acquired from CGGA database (Chinese Glioma Genome Atlas, http://cgga.org.cn/index.jsp)^45,46^.

### Estimate

The RNA-seq data from TCGA database were firstly transformed to transcripts per million (TPM) values. Then log2-scale transfermation was carried out for the RNA-seq data, followed which the RNA-seq data from TCGA database were merged with the microarray dataset (dataset ID: mRNA-array_301) from CGGA database. Normalization and batch effect correction for the merged data were conducted by using ‘sva’ package in R^47^. The immune, stromal, ESTIMATE score (the score representing the whole TME) and tumor purity for each LGG sample were calculated by ESTIMATE algorithm (Estimation of STromal and Immune cells in MAlignant Tumour tissues using Expression data) through the ‘estimate’ package in R^48^. The optimal cut-off value of the immune score was determined by the ‘survminer’ and ‘survival’ packages in R, based on which LGG samples were classified into high and low-immune score groups. The same method was utilized for the classification of LGG patients with distinct stromal scores.

### Identification of aging TME related signature

The robust DEGs between the low and high-immune score groups were determined with |log_2_ FC (fold change)|> 1 and adjusted p values (FDR, false discovery rate) < 0.001 by using the ‘limma’ package in R^49^. Similar method was utilized to screen out the robust DEGs between the low and high-stromal score groups. The DEGs above were further merged into one gene set which was defined as TME related gene signature (TMERS). WGCNA^50^ (‘WGCNA’ package in R) was used to determine the key genes significantly associated with aging TME based on the expression profiles of TMERS in the merged data. The soft-threshold values which ranged from 1 to 20, were examined based on the scale independence and mean connectivity degree of the co-expression network. The optimal soft-threshold value was determined when the scale independence was higher than 0.85 and the connectivity degree was relatively higher. The modules positively or negatively associated with aging TME (age and ESTIMATE score) were regarded as the key modules, the genes involved in which were identified as ATMERS.

### Classification for LGG samples based on the expression patterns of ATMERS

Firstly, univariate cox regression analysis was carried out to screen out the ATMERS with prognostic values in which p < 0.05 was regarded as statistically significant. The ‘ConsensusClusterPlus’ package in R^51^ was used to determine the optimal category of LGG samples based on the expression profiles of prognostic ATMERS. PCA was utilized to examine whether the LGG samples could be well distinguished based on the expression profiles of the prognostic ATMERS.

### Quantification of TME components

The relative abundance of the infiltrating immune cells in TME was evaluated based on the CIBERSORT algorithm^52^. Moreover, the abundance of several critical immune and stromal cells existing in TME were calculated through MCP counter^53^. TIDE website (http://tide.dfci.harvard.edu/) was employed to calculate the TIDE related scores for LGG samples to investigate the immunotherapeutic response.

### Aging TME scoring system

The genes involved in ATMERS which positively correlated with the prognosis of LGG patients were determined as favorable ATMERS and the genes which negatively correlated with prognosis were defined as unfavorable ATMERS. The enrichment score (GSVA score) regarding the two gene sets (unfavorable and favorable ATMERS) for each sample was calculated via GSVA method and single sample gene set enrichment analysis (ssGSEA) by using GSVA package in R software^54^. GSVA method serves as a popular method for scoring individual samples based on molecular characteristics or gene sets and acquired transcriptional data. GSVA represents a method that evaluates the enrichment of a specific function activity (negatively or positively associated with prognosis of LGG patients) over a sample population in an unsupervised manner. According to the method described by Hänzelmann et al.^54^, GSVA score of the unfavorable and favorable gene sets for each sample was calculated in our study. The aging TME score for each LGG sample was produced according to this formula: $agingTMEscore=\mathrm{GSVAscoreA}-\mathrm{GSVAscoreB}$, where the enrichment score for the unfavorable ATMERS was defined as GSVAscoreA and the enrichment score for the favorable ATMERS was defined as GSVAscoreB. The optimal cut-off value for the classification of LGG samples was determined by ‘survminer’ package in R based on which LGG samples were separated into the high and low-aging TME score groups.

### Construction of a nomogram model

A nomogram combining aging TME score and multiple clinicopathological factors was established to predict the prognosis of LGG patients more efficiently by using ‘rms’ package in R. The calibration curves, DCA and C-index were introduced to evaluate the predictive performance of the nomogram model.

### Functional enrichment analysis

The underlying molecular mechanisms between subgroups were explored using ‘GSVA’, ‘GSEABase’ and ‘limma’ packages in R, in which the reference files including “c5.go.mf.v7.4.symbols” and “c2.cp.kegg.v7.4.symbols” which were obtained from GSEA database were utilized. The terms with |log_2_ FC|> 0.1 and adjusted p values (FDR) < 0.05 were screened out. KEGG pathways and molecular functions involved in Gene Ontology (GO) terms were selected for the functional enrichment analysis.

### Somatic mutation analysis

The somatic mutation data (maf format) for LGG samples acquired through the whole exome sequencing platform were retrieved from TCGA database. The ‘maftools’ package in R was employed for the analysis and visualization of somatic variants. The cumulative nonsynonymous mutations in per million bases in coding regions were defined as TMBs.

### Validation of aging TME score in external data sets

A total of 172 LGG samples extracted from CGGA database (dataset ID: mRNAseq_325) were treated as a validation cohort^55,56^. A total of 110 LGG samples from GSE4271, GSE4412, GSE43378 and GSE84010^57–61^ were collected from the Gene Expression Omnibus database (GEO, https://www.ncbi.nlm.nih.gov/geo/). The gene expression profiles by array from GSE4271, GSE4412, GSE43378 and GSE84010 datasets were firstly log2-scale transformed and then normalization for the four datasets was carried out respectively for further analysis. Finally, the four datasets were merged into one dataset, in which batch effect correction was conducted by using ‘sva’ package in R^47^.The merged data set was treated as another validation cohort. In addition, the performance of aging TME score for predicting the immunotherapeutic response was verified by using IMvigor210 cohort according to the Creative Commons 3.0 License^62^.

### Validation of unfavorable ATMERS at protein level

Four genes including *CA3, HSPB6, FABP5* and *SERPINF1* were randomly selected from the unfavorable ATMERS. The expression patterns of the four genes in normal and LGG tissues were extracted from the Human Protein Atlas website (https://www.proteinatlas.org/)^63^.

Western blot analysis was carried out to further explore the expression levels of the four genes between normal and LGG tissues. Brain tissues obtained from epilepsy patients who received temporal lobe resection were treated as the control group. LGG tissues obtained from five patients who received tumor resection were treated as the tumor group.

Tissues were separately homogenized and lysed in cold RIPA lysis buffer containing PMSF and protease inhibitor at 0–4 °C for at least half hour. The lysates were then centrifuged at 1500*g* at 4 °C for 15 min to remove the debris. The protein in the supernatant was regulated to the same concentration by using Bio-Rad protein assay kit. Samples were homogenized with loading buffer containing SDS and then boiled at 100 °C for 5 min. Subsequently, equal amounts of protein samples were separately added to the 10% SDS-PAGE and electrophoresed at 60 V for 90 min. Afterwards, the separated protein samples on the SDS-PAGE were transferred to PVDF membranes at 50 V for 2 h. After incubation with the primary antibodies for 12 h at 4 °C, the PVDF membranes were then rinsed with PBS buffer for two times. The primary antibodies involved in the western blot analysis were as follows: anti-Carbonic Anhydrase 3 (CA3), anti-Hsp20 (HSPB6), anti-FABP5 and anti-PEDF (SERPINF1).The membranes were further incubated with the corresponding secondary HRP conjugated antibodies for two hours at 23 °C. After washed with PBS buffer for two times, protein bands at the specific locations were developed by ECL (enhanced chemiluminescence) solution. The images were visualized and saved by using ChemiDoc MP imaging system.

### Statistical analysis

Perl software (version 5.32.1.1) and R software (version 4.1.1) were utilized for the statistical analysis and visualization of the results. Chi-square tests were used to compare the categorical variables between subgroups and Student’s t-tests were utilized to compare the continuous data between subgroups. Two-sided p values < 0.05 were considered statistically significant if not specially noted. Kaplan–Meier survival analysis was used to compare the overall survival between two groups in which log-rank test was involved in the statistical analysis. The Receiver Operating Characteristic (ROC) curves were drawn via ‘survival’, ‘glmnet’, ‘survminer’ and ‘timeROC’ packages in R. Univariate and multivariate cox analysis were conducted by ‘survival’ R package.

### Ethics approval and consent to participate

This study has been approved by “Medical Ethics Committee of Qingdao Municipal Hospital”. We have obtained the approval and consent from the participates. We confirm that all experiments were performed in accordance with relevant named guidelines and regulations. We Confirm that informed consent was obtained from all participants.

## Supplementary Information

Supplementary Information.

## Data Availability

The datasets analyzed during the current study are available in TCGA database (The Cancer Genome Atlas, http://cancergenome.nih.gov/), CGGA database (Chinese Glioma Genome Atlas, http://cgga.org.cn/index.jsp/) and GEO database (Gene Expression Omnibus database, https://www.ncbi.nlm.nih.gov/geo/). Tissues used for western blot analysis were obtained from patients in department of neurosurgery, qingdao municipal hospital.
