# Supplementary material for: Characterization of aging tumor microenvironment with drawing implications in predicting the prognosis and immunotherapy response in low-grade gliomas
Source: Sci Rep. 2022 Mar 31;12:5457. doi: 10.1038/s41598-022-09549-3 (PMC8971489; doi:10.1038/s41598-022-09549-3)
Supplement: Supplementary file 1 — Supplementary Information. [file 41598_2022_9549_MOESM1_ESM.pdf]

Characterization of aging tumor microenvironment with drawing implications in predicting the prognosis and immunotherapy response in low-grade gliomas

Zijian Zhou, JinHong Wei, Wenbo Jiang

Supplementary table 1. Clinicopathological features of LGG patients in TCGA database

**Features**		**Total**	**High-aging TMEscore**	**Low-aging TMEscore**
**Age**	<45	300(59.06%)	26(28.57%)	274(65.71%)
	>=45	208(40.94%)	65(71.43%)	143(34.29%)
**Grade**	G2	246(48.52%)	18(19.78%)	228(54.81%)
	G3	261(51.48%)	73(80.22%)	188(45.19%)
**Histologic Type**	Astrocytoma	192(37.8%)	61(67.03%)	131(31.41%)
	Oligoastrocytoma	128(25.2%)	14(15.38%)	114(27.34%)
	Oligodendroglioma	188(37.01%)	16(17.58%)	172(41.25%)
**IDH1 Mutation**	Mutant	91(72.8%)	7(26.92%)	84(84.85%)
	Wildtype	34(27.2%)	19(73.08%)	15(15.15%)
**Gender**	Female	226(44.49%)	42(46.15%)	184(44.12%)
	Male	282(55.51%)	49(53.85%)	233(55.88%)
**Therapy Outcome**	Complete Remission/Response	83(35.93%)	9(21.95%)	74(38.95%)
	Partial Remission/Response	50(21.65%)	4(9.76%)	46(24.21%)
	Progressive Disease	39(16.88%)	18(43.9%)	21(11.05%)
	Stable Disease	59(25.54%)	10(24.39%)	49(25.79%)

Supplementary table 2. Clinicopathological features of LGG patients in CGGA database (DataSet ID: mRNA-array_301)

Features		Total	High-aging TMEscore	Low-aging TMEscore
**TCGA subtypes**	Classical	7(4.4%)	3(11.54%)	4(3.01%)
	Mesenchymal	31(19.5%)	18(69.23%)	13(9.77%)
	Neural	62(38.99%)	4(15.38%)	58(43.61%)
	Proneural	59(37.11%)	1(3.85%)	58(43.61%)
**Type**	Primary	143(89.94%)	22(84.62%)	121(90.98%)
	Recurrent	16(10.06%)	4(15.38%)	12(9.02%)
**Grade**	G2	106(66.67%)	11(42.31%)	95(71.43%)
	G3	53(33.33%)	15(57.69%)	38(28.57%)
**Gender**	Female	69(43.4%)	7(26.92%)	62(46.62%)
	Male	90(56.6%)	19(73.08%)	71(53.38%)
**Age**	<45	111(70.7%)	14(56%)	97(73.48%)
	>=45	46(29.3%)	11(44%)	35(26.52%)
**Radio status**	treated	136(86.62%)	21(80.77%)	115(87.79%)
	untreated	21(13.38%)	5(19.23%)	16(12.21%)
**Chemo status**	treated	69(44.81%)	20(76.92%)	49(38.28%)
	untreated	85(55.19%)	6(23.08%)	79(61.72%)
**IDH mutation**	Mutant	105(66.46%)	8(32%)	97(72.93%)
	Wildtype	53(33.54%)	17(68%)	36(27.07%)
**1p19q Codeletion**	Codel	16(32%)	0(0%)	16(38.1%)
	Non-codel	34(68%)	8(100%)	26(61.9%)
**MGMTp methylation**	methylated	43(28.67%)	6(26.09%)	37(29.13%)
	un-methylated	107(71.33%)	17(73.91%)	90(70.87%)

Supplementary table 3. 241 ATMERS genes
SRRM3
CRNDE
KCNB1
HRH3
PAK3
GFRA1
SEZ6L2
SPHKAP
CBLN1
DCX
CHRNB2
SEZ6L
FBLL1
SHANK2
CDK5R1
RTN1
CELSR3
GDAP1L1
BEX1
TNNT1
SCRT1
KCNK3
ST8SIA3
UNC5A
ATP6V1G2
CUX2
BRSK2
KCNQ2
SCN3B
NRXN1
NXPH1
DUSP26
ATCAY
SHISA7
SLC1A6
CDH18
TMEFF2
CRTAC1
SLC7A14
HECW1
CHRNA4
GNG4
KCNIP3
INA
GPRIN1
MMP24

TOX3
GRIN3A
REPS2
SEZ6
CA10
STX1B
B3GALT2
ACSL6
SSTR2
GPR17
C2orf80
GABRA3
PDE2A
MEGF11
VSTM2B
ALDOC
GLRA3
PSD
PTPRT
ADARB2
ELAVL4
SCAMP5
KCNJ11
DACH2
DOK6
OMG
L1CAM
DGCR5
SLC25A27
CSMD3
EPHA10
ELFN2
MAST1
FGF12
FAM110B
SMPD3
NMNAT2
TTC9B
SCG3
CPLX1
TMEM132B
PODXL2
CSMD1
UNC80
SLC6A1
BCL7A
CA3

DLGAP1
RUNDC3A
CADM2
MMD2
UNC13A
SLITRK1
CHGB
NOG
CACNG2
HIP1R
DSCAML1
MAPK8IP2
RIPPLY2
KSR2
JPH3
TNR
SNAP91
VSTM2A
ATP8A1
SPTBN2
ZDHHC22
MYT1
MAP2
NRSN1
NEUROD1
WASF1
LINGO1
SGSM1
SLC24A3
GABBR1
PID1
GABRB3
ABCC8
HTR1A
ENHO
RGR
FAM155A
TMEM196
GRIA2
ACTL6B
DGKB
RIMS2
SLIT1
WNT7B
SSTR1
NKAIN1
AP3B2

KCNIP2
ZFR2
PHYHIPL
CACNG4
INSM2
HSPB6
SLC17A8
MIAT
THBS1
LRRN1
PRLHR
MAOB
TMEM179
DLL1
KHDRBS2
DLL3
IL1R1
MRGPRF
C7
VASN
HPSE2
IL6
DDR2
PSD2
ITGA3
MAFB
ACBD7
HAPLN1
G0S2
C7orf57
RAMP3
PCDH15
FSTL5
MFAP4
SERPINF1
DSCAM
FERMT1
PPP1R1A
LGALS3
IL21R
VIPR2
SHD
NUDT11
FABP5
SFRP2
WDR38
NTSR2

TMEM100
RFTN1
HES5
NEU4
SOX8
ISLR
FAM183A
LRRC55
CSPG5
SLN
PDLIM4
RARRES2
KLRC2
IL1B
APLNR
HES6
IFITM1
FGFBP3
CNTFR
NCAN
SPHK1
AEBP1
FOSL2
SOD3
SMOC1
IGFN1
RPRM
LOXL1
TCTEX1D1
EFEMP2
OSM
FGFBP2
RASL10A
PODNL1
GRIA4
DDIT4L
GALNT13
DKK1
FMOD
TEKT1
AQP1
OLIG1
THBD
IL13RA2
BMP2
LHX5
SDC4

RPS4Y1
XIST
CH25H
EGR1
EGR2
SUSD5
CCL4

Supplementary table 4. Proportional hazard assumption test

	chisq	P value
**aging TME score**	2.08894	0.15
**Gender**	0.13321	0.72
**Age**	0.08123	0.78
**Grade**	1.87797	0.17
**IDH mutation**	0.00144	0.97
**Nomogram**	4.13177	0.53

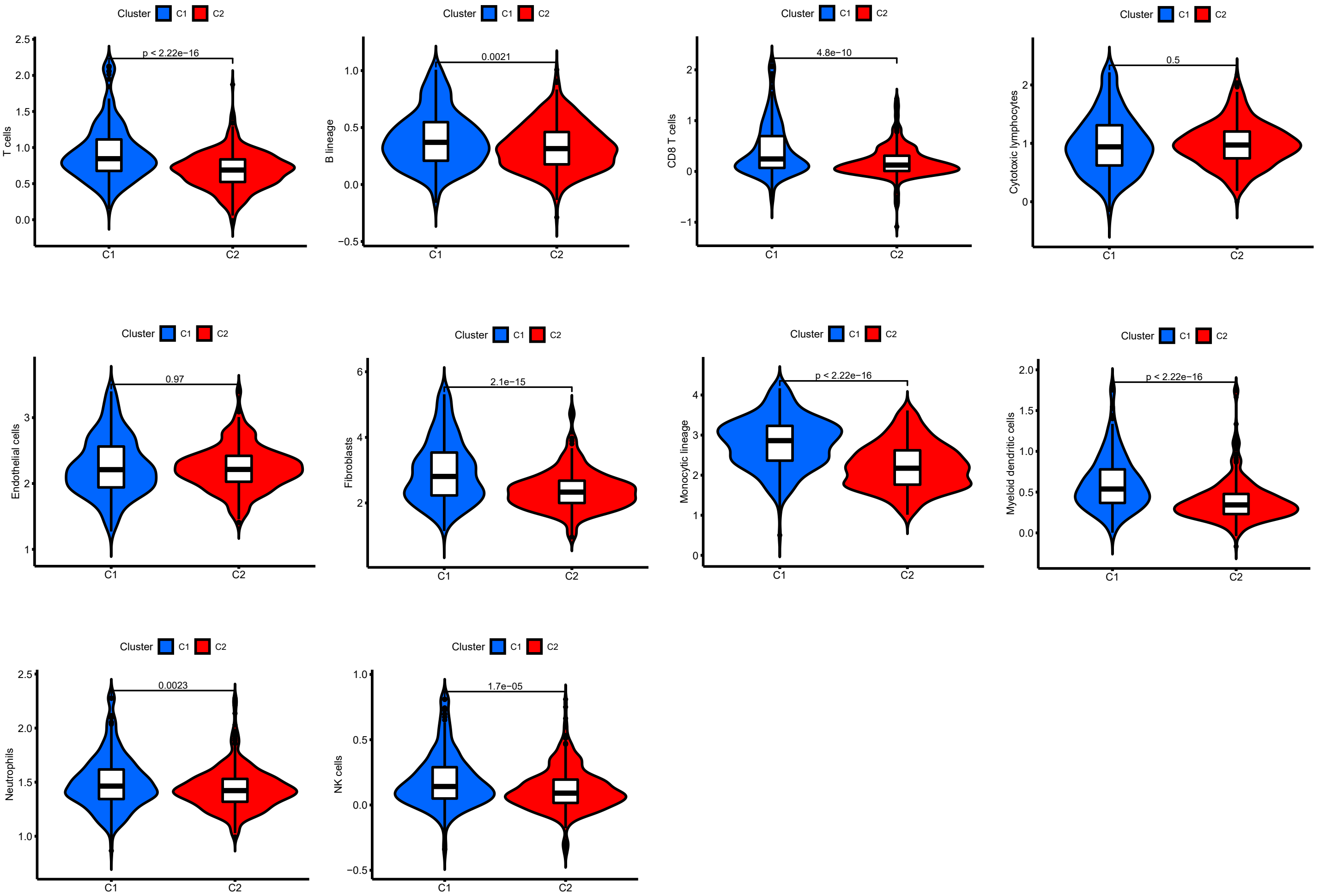

Supplementary figure 1. Comparisons of TME components between two clusters. TME, tumor microenvironment.

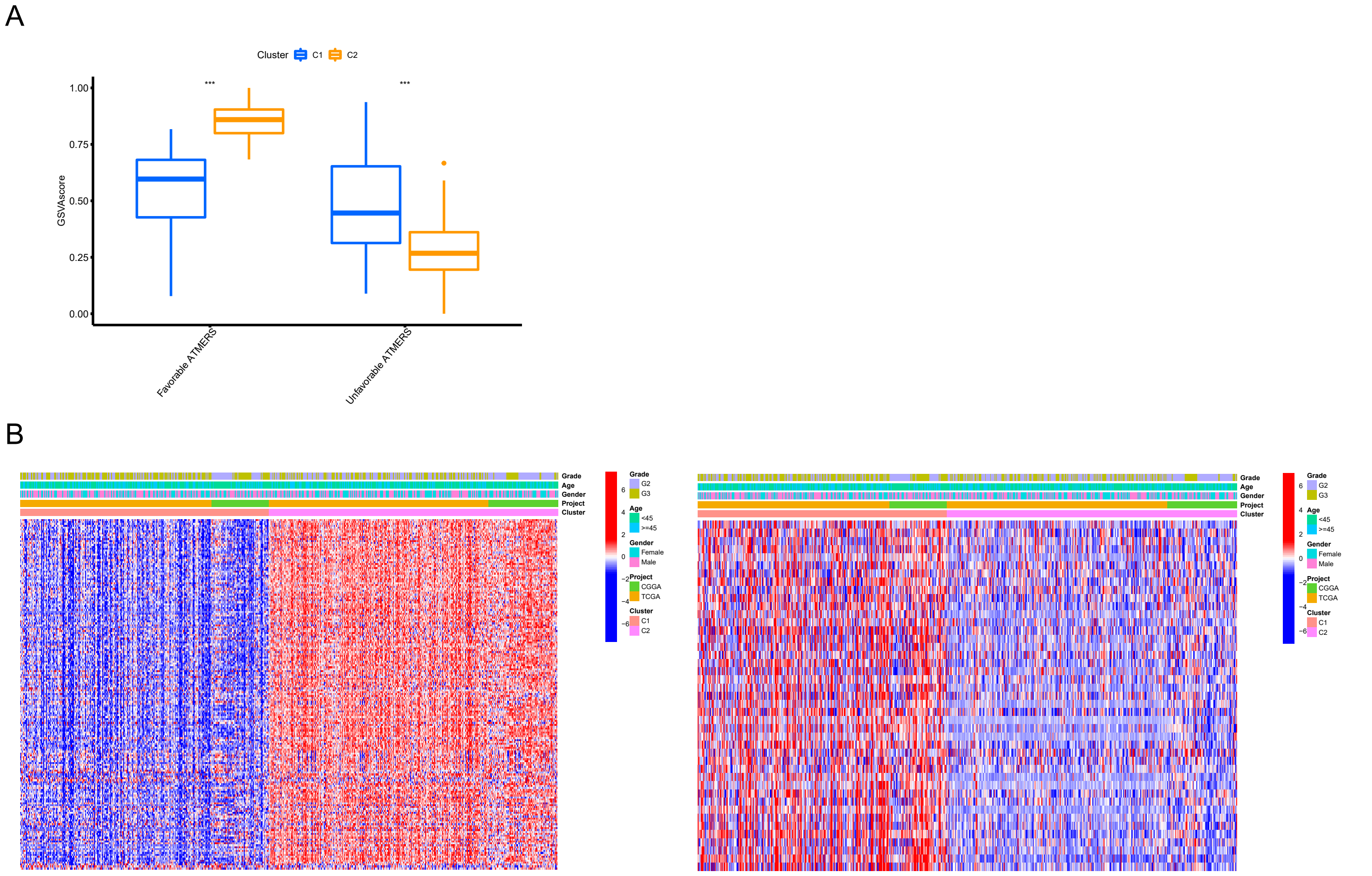

Supplementary figure 2. (A) Comparisons of GSVA scores for favorable/unfavorable ATMERS between two clusters. The expression patterns of favorable (left panel) and unfavorable (right panel) ATMERS between two clusters. GSVA, gene set variation analysis; ATMERS, aging tumor microenvironment related signature; ***means p <0.001.

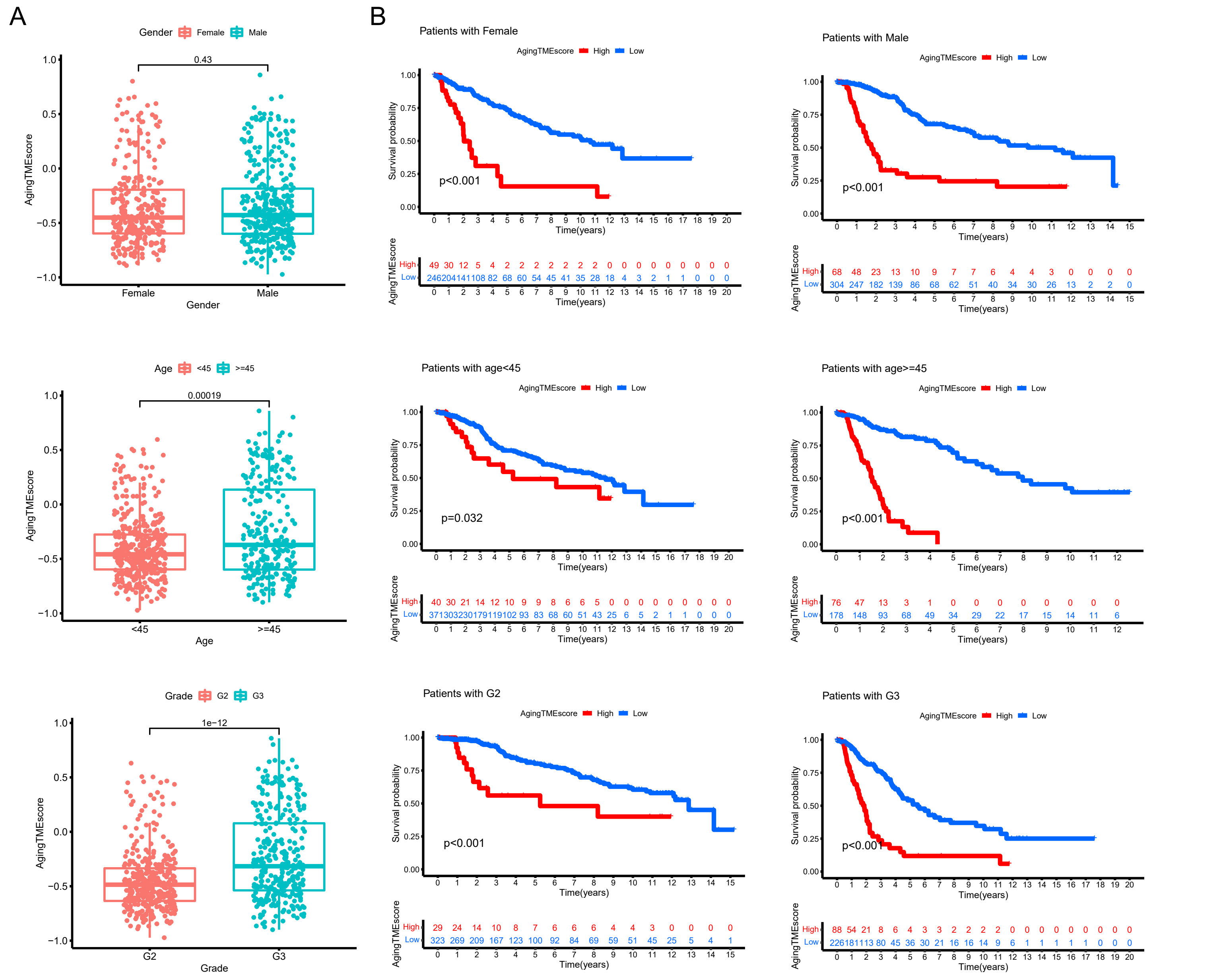

Supplementary figure 3. (A) Comparisons of aging TME scores between LGG patients with different clinical features. (B) Kaplan–Meier survival analysis of low and high aging TME score groups with different clinical features. TME, tumor microenvironment.

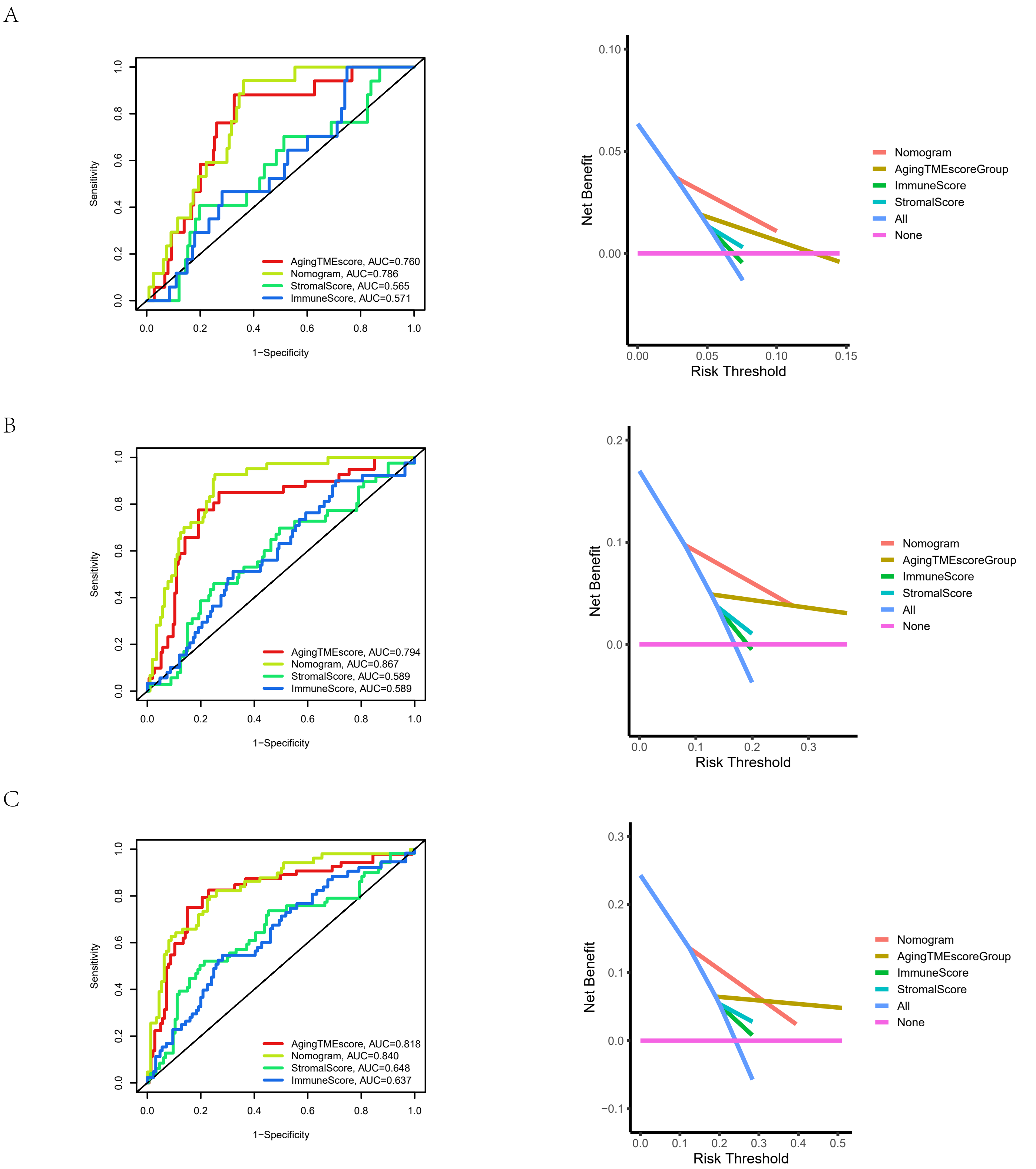

Supplementary figure 4. (A) The ROC curves of the nomogram model for predicting the 1-year overall survival (left panel) and the DCA of the nomogram model for predicting the 1-year overall survival (right panel) in LGGs, compared to immune and stromal score. (B, C) The similar results predicting the 2 and 3-year overall survival in LGGs. ROC, receiver operating characteristic; AUC, area under curves; DCA, decision curve analysis.

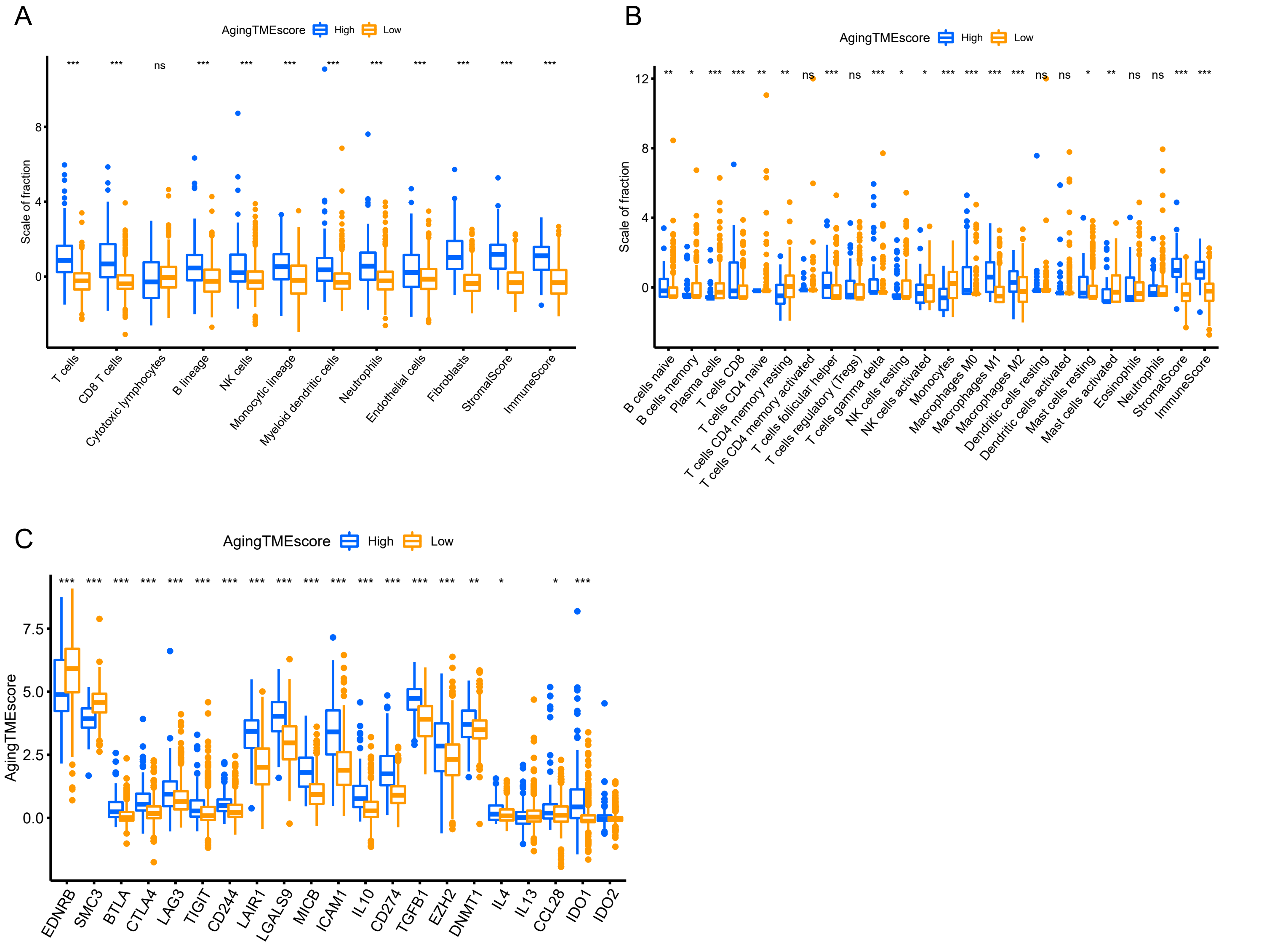

Supplementary figure 5. (A) Comparisons of TME components between two groups by using MCP counter algorithm. (B) Comparisons of infiltrating immune cells between two groups by using CIBERSORT algorithm. (C) Comparisons of expression levels for genes involved in the negative regulation of anti-tumor immune response between two groups. * means $p < 0.05$, ** means $p < 0.01$ and ***means $p < 0.001$. TME, tumor microenvironment.

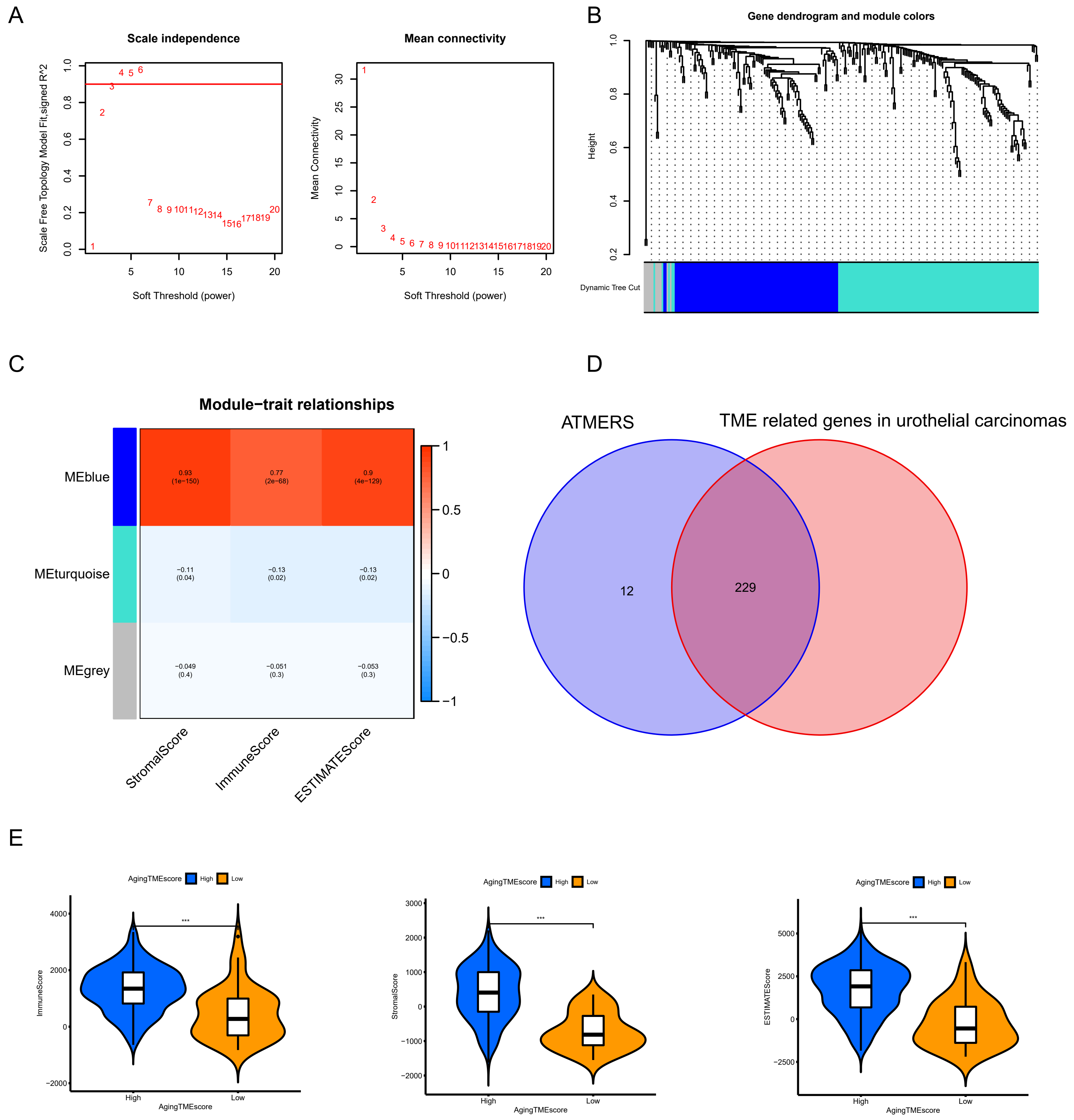

Supplementary figure 6. The TME of LGG shared common features with those of urothelial carcinoma (IMvigor210 cohort) in the expression patterns of TME related genes. (A) Determination of the scale-independence degree (left) and the mean connectivity index (right) when the optimal soft-threshold value (power value) was set to 3. (B) Dendrogram of all ATMERS and modules with different colors. (C) Heatmap showing the key modules which mostly correlated with the scores with respect to TME in urothelial carcinoma (IMvigor210 cohort). The Pearson correlation coefficients and p values were displayed in cells. (D) 229 out of 241 genes in the ATMERS significantly correlated with the characteristics of TME in urothelial carcinoma (IMvigor210 cohort). (E) The high aging TME score group exhibited significantly higher scores with respect to TME compared to the low aging TME score group in urothelial carcinoma (IMvigor210 cohort). TME, tumor microenvironment; LGG, low-grade glioma; ATMERS, aging tumor microenvironment related signature; * means $p < 0.05$, ** means $p < 0.01$, and ***means $p < 0.001$.

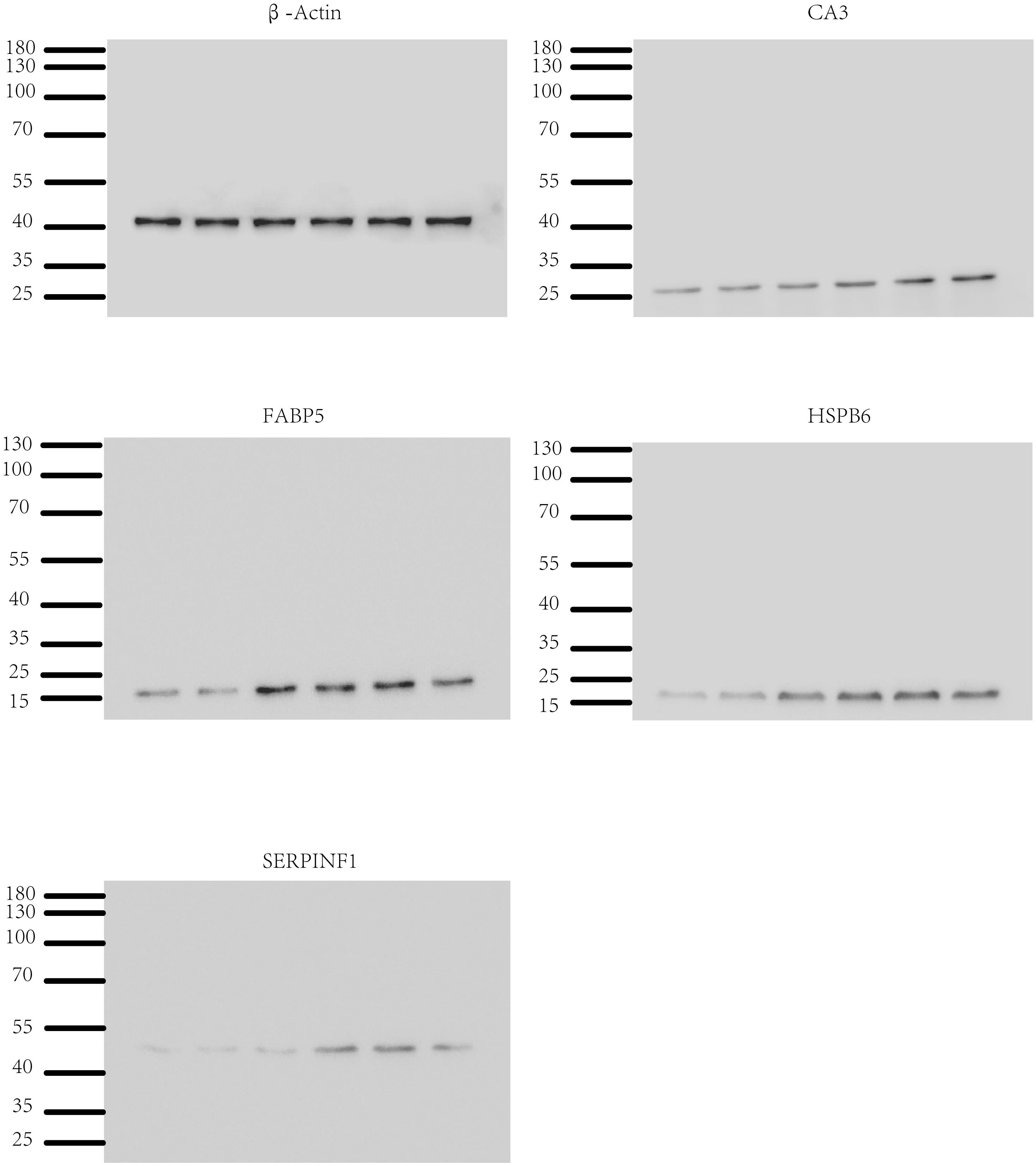

Supplementary figure 7. The uncropped original images of western blotting analysis.

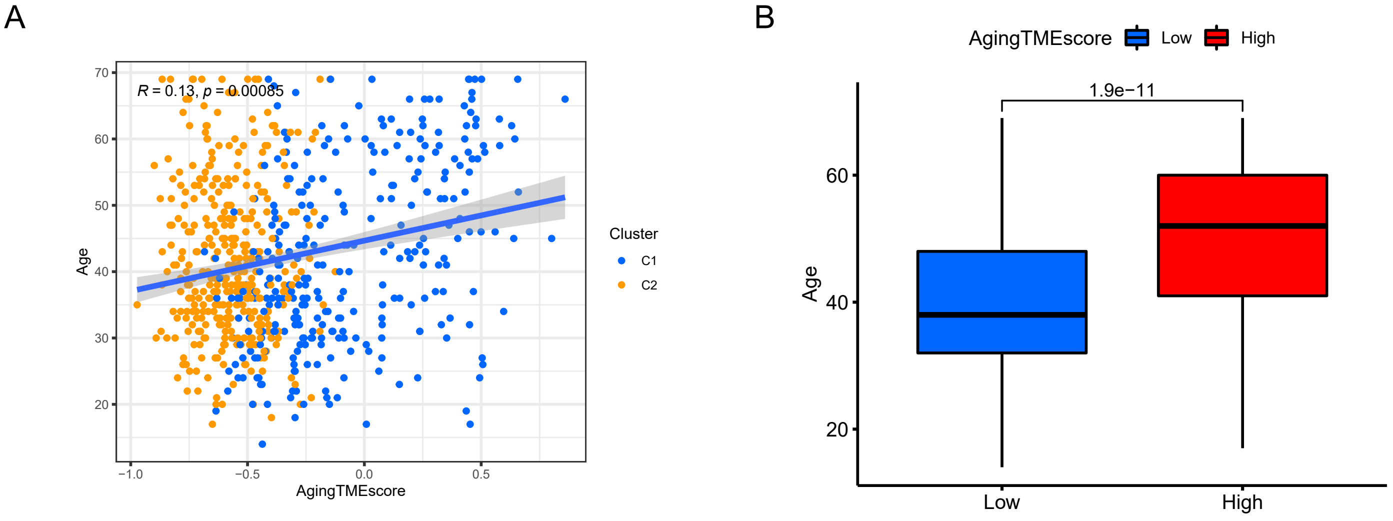

Supplementary figure 8. (A) Correlation analysis between aging TME score and age. (B) Comparison of age between two groups. TME, tumor microenvironment.

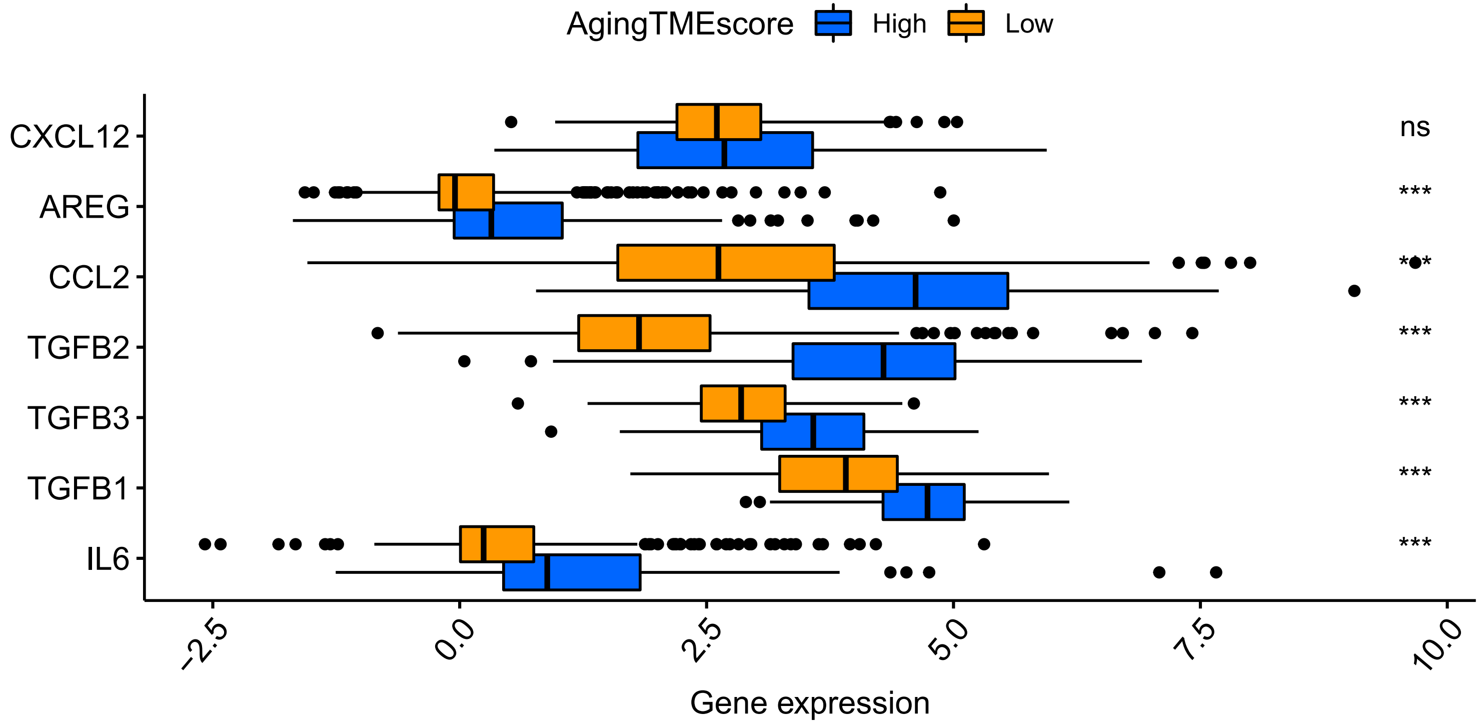

Supplementary figure 9. Comparisons of the expression levels of the related genes between two groups. TME, tumor microenvironment; ***means p <0.001.
